# Case Report of Acute Central Airway Obstruction Induced by Anti‐PD‐1 Sintilimab: Clinical Presentation and Review

**DOI:** 10.1002/ccr3.71811

**Published:** 2026-01-02

**Authors:** Ting Ouyang, Zeqiang Wang, Weidong Zhang, Wei Liu

**Affiliations:** ^1^ Department of Respiratory and Critical Care Medicine Hunan Provincial People's Hospital/the First Affiliated Hospital of Hunan Normal University Changsha Hunan China

**Keywords:** bronchoscopy, case report, central airway obstruction, immune‐related adverse events, PD‐1 inhibitor, Sintilimab

## Abstract

Immune checkpoint inhibitors (ICIs), such as sintilimab, have revolutionized non‐small cell lung cancer (NSCLC) treatment but can trigger immune‐related adverse events (irAEs). While pneumonitis is well documented, central airway obstruction (CAO) due to immune‐mediated necrosis is an extremely rare and life‐threatening phenotype that poses significant diagnostic challenges. A patient with recurrent squamous cell carcinoma of the lung achieved partial remission after two cycles of sintilimab combined with chemotherapy. However, 48 h after the third cycle, the patient developed acute, severe dyspnea and hypoxemia. Emergency bronchoscopy revealed extensive necrotic material occluding the right main bronchus and carina. Pathological analysis indicated lymphocytic infiltration with necrosis, while microbiological tests were negative for pathogens. Given the temporal relationship, exclusion of alternative etiologies, and response to corticosteroids, the event was considered *probable* sintilimab‐related acute necrotizing CAO. Immediate interventional bronchoscopy with cryoablation was performed to restore airway patency. This was followed by a short course of systemic corticosteroids (methylprednisolone, then a prednisone taper). The patient's symptoms resolved completely, and follow‐up bronchoscopies confirmed mucosal healing without recurrence of stenosis or necrosis. This case highlights a distinct, underrecognized pulmonary irAE manifesting as acute central airway necrosis. Although a definitive causal relationship cannot be established from a single case, the clinical course strongly suggests a probable association with sintilimab.

## Key Clinical Message

Sintilimab may potentially cause life‐threatening, acute necrotizing central airway obstruction. Clinicians should be aware that this complication requires immediate bronchoscopic cryoablation followed by corticosteroids.

## 1 Introduction

Immune checkpoint inhibitors (ICIs) restore T cell‐mediated anti‐tumor immunity by blocking inhibitory pathways such as PD‐1/PD‐L1 or CTLA‐4, significantly improving survival in various malignancies, including non‐small cell lung cancer (NSCLC) [1]. As a domestic PD‐1 inhibitor, sintilimab has demonstrated efficacy in first‐line combination chemotherapy for squamous NSCLC (ORIENT‐12) [2].

However, while enhancing immune responses, ICIs can also trigger immune‐related adverse events (irAEs) affecting multiple organs. The manifestations and severity of irAEs are highly heterogeneous [3]. Standard management includes discontinuation of suspected drugs, systemic corticosteroids, and targeted immunosuppressants or biologics for steroid‐refractory cases. Given differences in immune mechanisms and dynamics across organs, irAE management should be organ‐specific and multidisciplinary [3].

The respiratory system is commonly affected by irAEs, though the existing literature primarily focuses on parenchymal or small‐airway damage (e.g., pneumonitis). In contrast, central airway obstruction (CAO), caused by tumors, benign stenosis, or other factors, is a life‐threatening thoracic emergency that can rapidly lead to respiratory failure [4]. Although severe ICI‐related irAEs such as myocarditis, neuropathy, and relapsing polychondritis have been reported [5, 6, 7], inflammatory or necrotizing CAO primarily involving central airways remains extremely rare. Its imaging and bronchoscopic features differ significantly from those of classic immune‐related pneumonitis, and emergency management strategies vary, making clinical recognition and diagnosis challenging.

Currently, detailed case reports of PD‐1 inhibitors directly causing CAO are lacking. Here, we describe the clinical and bronchoscopic characteristics of such a case, its successful management via airway cryoablation and short‐course corticosteroids, and how it differs from classic immune‐related pneumonitis.

## 2 Case Presentation

### 2.1 Case History

An adult patient with a history of squamous cell carcinoma of the right upper lung (cT2N1M0, stage IIIA) underwent right upper lobectomy 6 years ago. The patient declined adjuvant chemotherapy postoperatively. Four years ago, local recurrence was treated with palliative radiotherapy (60 Gy/30 fractions). Three years ago, the patient was diagnosed with and cured of right‐sided tuberculous pleuritis after standard anti‐tuberculosis therapy.

**FIGURE 1:**
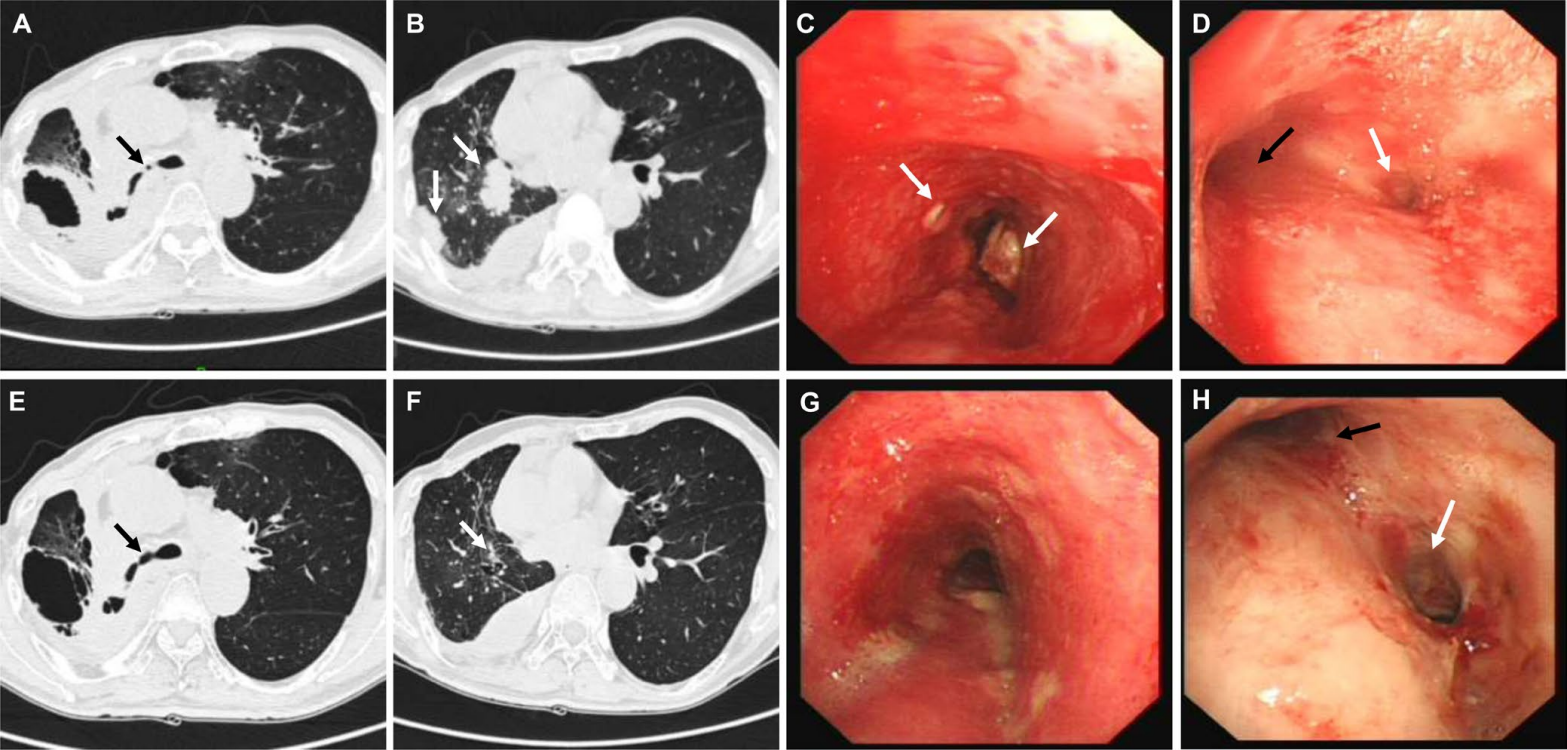
Comparison of chest CT and bronchoscopic findings before treatment and after two cycles of sintilimab combined with chemotherapy. (A) Baseline chest CT shows a cavitary lesion in the right lung (white arrow) and severe stenosis of the right main bronchus (black arrow). (B) Baseline CT reveals multiple nodular lesions in the right lung (white arrow). (C) Pre‐treatment bronchoscopy shows granular neoplasms in the lower trachea with mucosal congestion and edema (white arrow). (D) Pre‐treatment endoscopic findings: Left main bronchus appears normal (black arrow), while the right main bronchus shows severe stenosis with localized scarring (white arrow). (E) Post‐treatment CT shows a reduction in the cavitary lesion in the right lung and improvement in the lumen of the right main bronchus. (F) Post‐treatment CT shows significant absorption of the right lung nodules (white arrow). (G) Post‐treatment bronchoscopy shows regression of the neoplasms in the lower trachea, with reduced mucosal swelling. (H) Post‐treatment endoscopy shows a normal left main bronchus (black arrow), expansion of the lumen of the right main bronchus, smooth mucosa, and residual scarring (white arrow).

Two months before this hospitalization, a chest CT scan revealed a cavitary lesion with multiple nodules in the right lung, and diffuse nodular thickening of the right main bronchial wall with severe stenosis of the lumen (Figure 1A,B). Bronchoscopy showed extensive granular neoplasms in the lower trachea with significant mucosal congestion and swelling. The narrowest point of the right main bronchus had a diameter of approximately 4 mm, with localized scarring observed (Figure 1C,D). Biopsy pathology confirmed the recurrence of squamous cell carcinoma, but the patient refused PD‐L1 expression and driver gene testing.

### 2.2 Differential Diagnosis, Investigations, and Treatment

Based on the diagnosis of recurrent squamous carcinoma, the patient was started on an immune‐based chemotherapy regimen: sintilimab (200 mg) combined with liposomal paclitaxel (210 mg) and cisplatin (120 mg) in a 21‐day cycle. After completing two cycles of treatment, the patient was evaluated before the third cycle. CT showed a 66% reduction in the target lesion diameter compared to baseline, and according to RECIST 1.1 criteria, partial remission was achieved. Bronchoscopy revealed regression of the granular neoplasms in the lower trachea, with reduced mucosal congestion and swelling. The carina and right main bronchial mucosa appeared smooth, and the lumen diameter was restored to approximately 8 mm, with residual scarring (Figure 1E–H).

**FIGURE 2:**
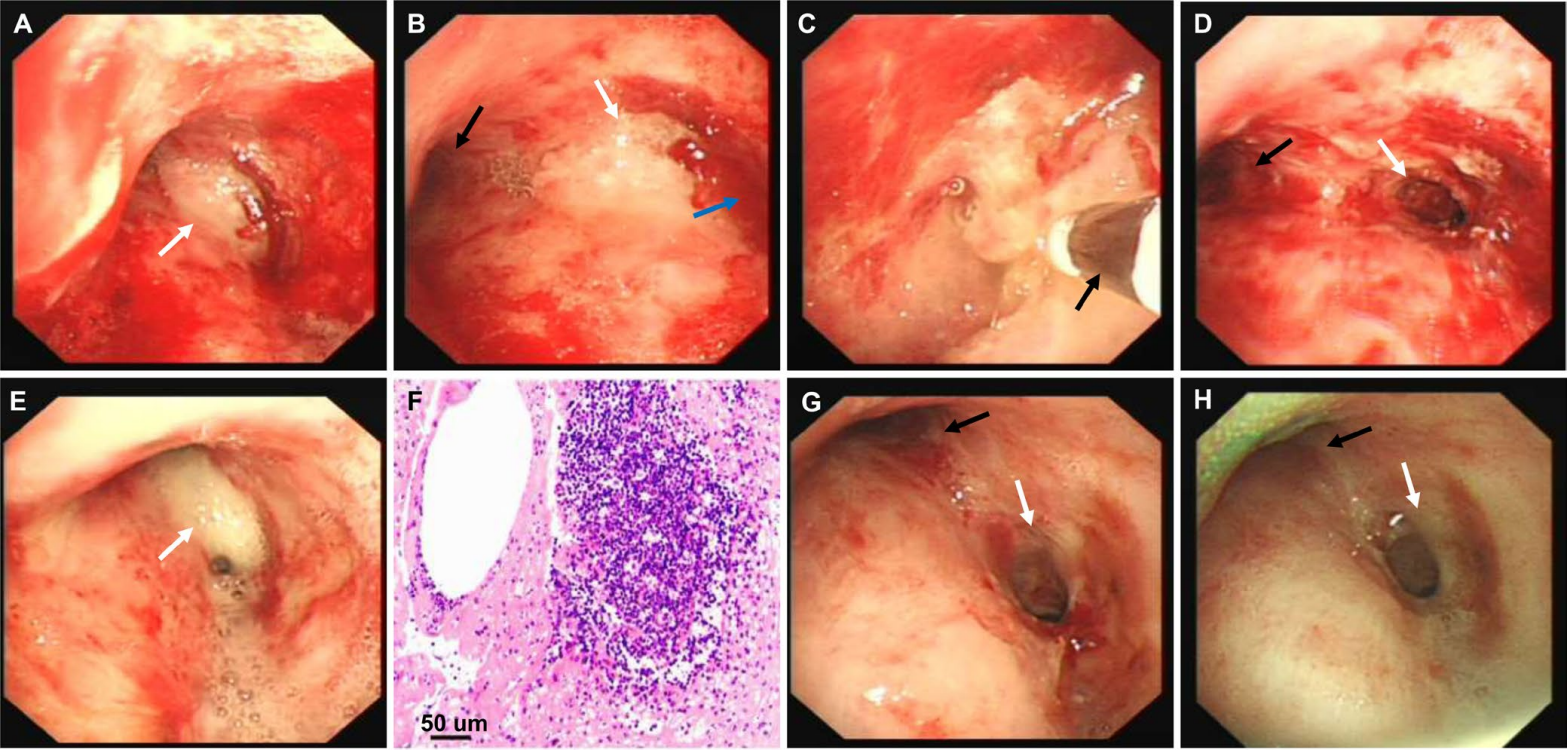
Bronchoscopic findings at the onset of the adverse event after the third cycle of immunotherapy and during follow‐up. (A) Diffuse hyperemia and edema of the distal tracheal mucosa, with abundant necrotic material adherent to the carina and right main bronchus (white arrows). (B) Necrotic material at the carina (white arrow); complete occlusion of the right main bronchial lumen by necrotic debris (blue arrow); left main bronchus remains patent (black arrow). (C) Bronchoscopic cryo‐extraction of necrotic material; the cryoprobe is visible (black arrow). (D) After cryotherapy, luminal patency of the right main bronchus is improved (white arrow). (E) On the following day, large amounts of necrotic material persist at the carina and in the right main bronchus (white arrows). (F) Histopathology showing focal lymphocytic infiltration on a background of inflammatory necrosis (Hematoxylin–Eosin stain; scale bar: 50 μm). (G) Re‐evaluation after 5 days of corticosteroid therapy: Left main bronchus patent with smooth mucosa (black arrow); right main bronchus patent with smooth mucosa and focal scarring (white arrow). (H) Four‐month follow‐up: Left main bronchus remains patent with smooth mucosa (black arrow); right main bronchus maintains patency with smooth mucosa and localized scarring (white arrow).

However, approximately 48 h after receiving the third cycle of sintilimab infusion, the patient developed sudden, acute, progressive dyspnea, with oxygen saturation dropping to 78%. Physical examination revealed the three depression signs with wheezing bilaterally, highly suggestive of central airway obstruction (CAO). Emergency bronchoscopy revealed a large amount of necrotic material occluding the carina and the right main bronchial lumen, leading to severe CAO (Figure 2A–D). Bronchoscopic cryotherapy was performed, and the airway was restored to patency. A follow‐up bronchoscopy the next day still showed residual necrotic material, prompting a second round of cryotherapy (Figure 2E).

Differential diagnosis primarily focused on infection versus tumor progression. Investigations included bronchoalveolar lavage fluid sent for second‐generation sequencing (mNGS), which did not detect any pathogens, and bacterial, fungal cultures, and acid‐fast staining were all negative. Pathological examination indicated inflammatory exudation and necrosis with multifocal lymphocytic infiltration, with no malignant tumor cells observed (Figure 2F). After ruling out other causes, the clinical team considered the event most consistent with a probable immune therapy‐related CAO.

Considering the patient's prior history of tuberculosis and to cautiously manage potential infection risks, methylprednisolone 40 mg/day was administered intravenously for 5 days, followed by oral prednisone 30 mg/day, with a weekly dose reduction of 5–10 mg, and treatment was discontinued after a total of 4 weeks. Supportive therapy, including high‐flow oxygen therapy, inhaled corticosteroids, and bronchodilators, was also provided.

### 2.3 Outcome and Follow‐Up

On day 5 after initiating steroid treatment, a follow‐up bronchoscopy showed complete resolution of the necrotic material and smooth mucosa (Figure 2G). The patient's dyspnea completely resolved, and they were successfully weaned off oxygen and discharged. At the patient's request, further immunohistochemical analysis and imaging follow‐up were declined, and anti‐tumor treatment was discontinued in favor of best supportive care.

**FIGURE 3:**
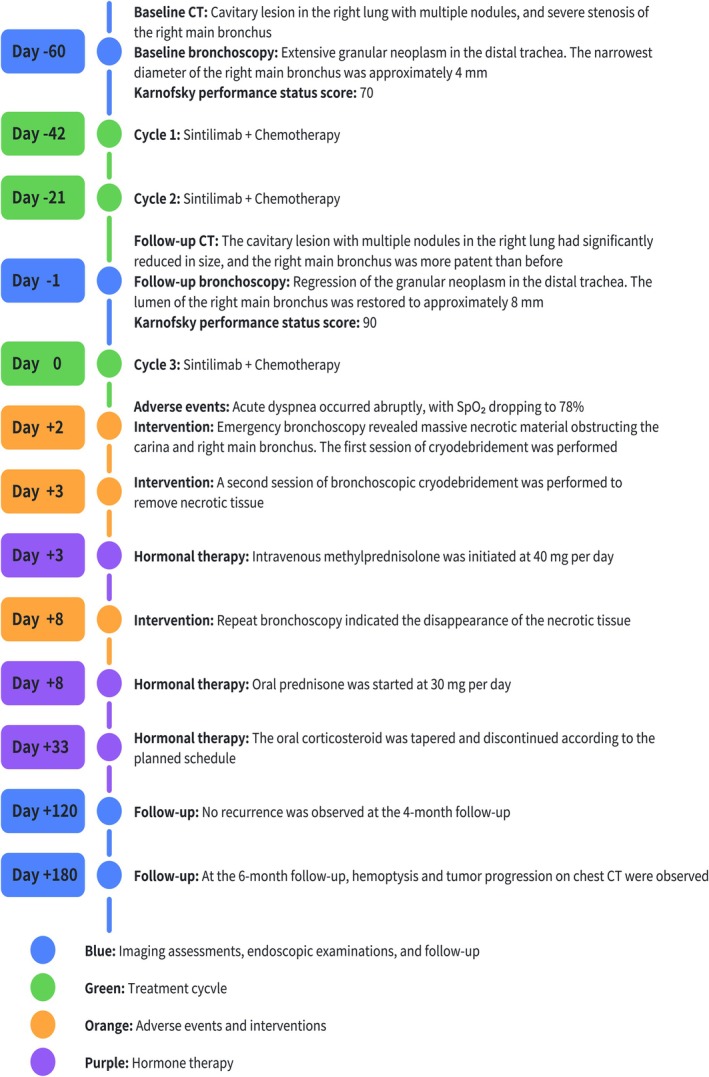
Schematic timeline of key clinical events during the diagnosis and treatment of this patient.

During the subsequent 4‐month period, two follow‐up bronchoscopies showed no recurrence of necrotic material or airway stenosis (Figure 2H) and no further respiratory symptoms occurred. The patient's ability to perform daily activities returned to the level prior to the adverse event. Unfortunately, at the 6‐month follow‐up, the patient presented with hemoptysis and was found to have tumor progression on a chest CT scan performed at an outside institution. Figure 3 illustrates the timeline of key events during the patient's diagnosis and treatment process, demonstrating successful management of immune therapy‐related CAO with bronchoscopic intervention and corticosteroids, leading to sustained clinical improvement.

## 3 Discussion

This case report describes a novel irAE probably associated with sintilimab: acute necrotizing central airway obstruction. The patient developed acute airway obstruction within 48 h of treatment, despite imaging and bronchoscopy showing treatment efficacy, with significant necrotic material blocking the airway. After bronchoscopy and corticosteroid therapy, symptoms improved quickly, with no recurrence during follow‐up. This differs fundamentally from classic immune‐related pneumonitis, suggesting that airway‐dominant irAEs must be considered in the differential diagnosis of thoracic emergencies during immunotherapy.

In contrast to previously reported airway lesions like tracheobronchial chondritis [7], this case presents a novel phenotype characterized by acute necrotizing mechanical obstruction. Histopathology showed extensive lymphocytic infiltration and necrosis, suggesting a possible mechanism driven by T‐cell overactivation. By blocking checkpoints like PD‐1/PD‐L1, ICIs induce clonal expansion of effector T cells [8]. These cells may destroy airway epithelium through cytotoxic molecules (e.g., perforin, granzyme) or the Fas–FasL death receptor pathway [9]. Additionally, the reactivation of tissue‐resident memory T cells (Trm), a mechanism observed in ICI‐induced colitis [10], might have triggered local necrosis in the airway mucosa. While humoral immunity and autoantibodies are involved in other irAEs [11], our pathological findings—necrosis with lymphocytic infiltration—primarily support a T‐cell‐mediated acute immune attack. Moreover, the patient's history of tuberculosis and thoracic radiotherapy likely contributed to the severity of the event. Previous tissue damage may have altered the local microenvironment or enhanced the immune response via “epitope spreading” [12, 13]. These factors could have promoted the rapid formation and retention of necrotic material. It is important to note that specific immunohistochemical staining (e.g., for CD4, CD8, or PD‐L1) and cytokine analysis were not performed; thus, our mechanistic explanation remains a plausible hypothesis based on morphological features and clinical context.

Clinically, distinguishing acute necrotizing CAO from classic immune‐related pneumonitis (CIP) is critical for survival. CIP primarily affects the lung parenchyma, typically presenting with non‐specific symptoms such as dry cough and progressive exertional dyspnea. Radiographically, CIP is characterized by diffuse ground‐glass opacities, consolidations, or interlobular septal thickening [14]. Conversely, ICI‐associated CAO is a mechanical emergency. Its clinical hallmark is the rapid onset of inspiratory dyspnea, audible stridor, and suprasternal/supraclavicular retractions (“three depression signs”), reflecting upper airway compromise rather than alveolar gas exchange failure. Therefore, when a patient on ICIs presents with acute respiratory distress, clinicians should not rely solely on lung window CT findings; airway patency must be specifically evaluated.

Acute CAO has diverse etiologies and requires a systematic differential diagnosis. First, tumor progression often presents as enlargement of existing lesions or the appearance of new lesions. However, in this case, imaging showed a significant reduction in tumor burden, and bronchoscopic biopsy did not detect malignant cells, thus excluding this possibility. Secondly, we excluded infectious etiologies. Microbial cultures, metagenomic Next‐Generation Sequencing (mNGS), and serological markers were all negative. Furthermore, pathology showed no evidence of infection. While tuberculosis recurrence and radiation necrosis were considered, neither fit the clinical picture. Tuberculosis recurrence is typically subacute or chronic, contrasting with this patient's rapid deterioration within 48 h. Similarly, radiation‐induced damage usually manifests as fibrosis months after therapy, which does not align with the acute necrotic features observed here. Additionally, mucus plugs are commonly seen in patients with asthma or chronic airway diseases and usually appear as gelatinous tubular structures on bronchoscopy, which were not observed in this case, thus excluding this possibility. After systematic exclusion, ICI‐related acute necrotizing CAO was deemed the most likely diagnosis and was subsequently confirmed by the rapid response to immunosuppression.

Existing literature on PD‐1 inhibitor–related airway lesions is largely limited to cases of nivolumab‐associated tracheobronchial chondritis [7, 15]. These cases typically manifest as subacute or chronic inflammation characterized by bronchial wall thickening, cartilage loss, and potential anti–type II collagen autoimmunity. In stark contrast, the current case defines a distinct, high‐acuity phenotype: sintilimab‐associated necrotizing central airway obstruction. Unlike the chronic inflammatory or chondritic patterns previously reported, our patient presented within 48 h with massive mucosal necrosis and mechanical blockage, requiring immediate bronchoscopic cryodebridement rather than relying solely on steroid tapering. This distinction suggests a different underlying immunopathology, potentially driven by acute T‐cell–mediated cytotoxicity against the airway epithelium rather than a humoral autoimmune response against cartilage.

Unlike the management of pneumonia‐type irAEs, CAO should adopt a dual‐track strategy of “intervention for recanalization first, followed by immunosuppression.” For patients with established ventilation obstruction, immediate bronchoscopic intervention should be performed (such as cryoablation, thromboembolization, balloon dilation, or stent placement). After excluding infection, corticosteroids (such as methylprednisolone 1–2 mg/kg/day) should be administered early and at adequate doses. In cases of refractory or recurrent symptoms, other immunosuppressive agents can be added. In this case, intervention combined with moderate‐dose corticosteroids successfully controlled the condition, providing clinical evidence for this approach.

This study has certain limitations. As a case report, it did not include immunohistochemical analysis (such as CD4^+^, CD8^+^ T cells, B cell, or macrophage subpopulation analysis), T cell receptor sequencing, or cytokine profiling, and therefore the predominant immune cell subsets and key signaling pathways could not be clearly identified. Future prospective studies are needed to further define the incidence, risk factors, mechanisms, and optimal treatment strategies for this type of irAE, providing a basis for clinical individualized prevention and treatment.

## 4 Conclusion

Probable sintilimab‐related acute necrotizing CAO is a rare but potentially fatal irAE, whose mechanisms may involve T cell cytotoxicity or local immune microenvironment alterations caused by prior tuberculosis or radiotherapy. This case underscores the need for heightened vigilance regarding airway involvement in patients receiving PD‐1 inhibitors. Early recognition, timely intervention, and short‐course immunosuppression are critical for effective management. Incorporating such airway emergencies into irAE monitoring and treatment pathways may enhance clinical responsiveness and improve patient outcomes.

## Author Contributions

**Ting Ouyang:** investigation, writing – original draft, writing – review and editing. **Zeqiang Wang:** writing – original draft. **Weidong Zhang:** writing – original draft. **Wei Liu:** investigation, supervision, writing – review and editing.

## Funding

The authors have nothing to report.

## Ethics Statement

In accordance with our institutional policy, case reports are exempt from Institutional Review Board approval. Nevertheless, formal written consent was obtained from the patient.

## Consent

Written informed consent was obtained from the patient for publication of this case report.

## Conflicts of Interest

The authors declare no conflicts of interest.

## Data Availability

The data that support the ﬁndings of this study are available from the corresponding author upon reasonable request.
